# Exotic Snakebites Reported to Pennsylvania Poison Control Centers: Lessons Learned on the Demographics, Clinical Effects, and Treatment of These Cases

**DOI:** 10.3390/toxins12120755

**Published:** 2020-11-29

**Authors:** Stephen W. Miller, Kevin C. Osterhoudt, Amanda S. Korenoski, Ketan Patel, Sakthivel Vaiyapuri

**Affiliations:** 1 School of Pharmacy, University of Reading, Reading RG6 6UB, UK; 2 The Poison Control Center, Children’s Hospital of Philadelphia, Philadelphia, PA 19104, USA; osterhoudtk@email.chop.edu; 3 Pittsburgh Poison Center, University of Pittsburgh, Pittsburgh, PA 15213, USA; johnsonas@upmc.edu; 4 School of Biological Sciences, University of Reading, Reading RG6 6UB, UK; ketan.patel@reading.ac.uk

**Keywords:** snakebite, envenomation, exotic, non-native, United States, poison center, antivenom

## Abstract

Exotic snakebites (i.e. from non-native species) are a rare occurrence, but they present a unique challenge to clinicians treating these patients. Poison control centers are often contacted to assist in the management and care of these medical emergencies. In this study, we analyzed case records of the two Pennsylvania poison control centers from 2004 to 2018 to describe clinical features reported as a result of exotic snakebite envenomation. For the 15-year period reviewed, 18 exotic snakebites were reported with effects ranging from mild local tissue injury to patients who were treated with mechanical ventilation due to respiratory failure. The mean age of the patients was 35 years and males accounted for 83% of the cases. Antivenom, the only specific treatment, was administered in seven of 18 patients within an average of four h of envenomation. The procurement of antivenom against these exotic species may require substantial logistical efforts due to limited stocking of this rarely used treatment. Newer, targeted, small molecule treatments that are being currently investigated may aid in the treatment of snakebites in general. However, people should be cautious when handling these exotic species, and clinicians should be aware of these bites and relevant clinical effects in order to manage these when reported.

## 1. Introduction

While fear of snakebites is relatively common, fatalities from snakebite envenomation (SBE) are a rare occurrence in industrialized countries due to prompt access to appropriate medical care. Globally, estimates of annual fatalities range from 80,000 to over 120,000 are likely to be underestimated due to a lack of reporting in the rural areas of less developed countries where snakebites are most prevalent [1,2]. Most of the SBE occur in rural areas of South and Southeast Asia, Sub-Saharan Africa, Oceania, and South America [3,4,5]. The access to medical facilities is limited in these regions. However, in the United States of America (USA), venomous snakebites occur primarily in the Southern and Western states with annual estimates ranging from a few thousand to 6000 or more resulting in less than 20 fatalities [6,7]. The vast majority of these bites are caused by viperid species native to North America and consist of rattlesnakes (*Crotalus* spp.), copperheads, cottonmouths (*Agkistrodon* spp.), and a small number of elapid coral snakes (*Micrurus* spp.) [8]. In the USA., exotic, non-native species are estimated to cause over 30 SBE per year [9,10]. The trade in venomous snakes increases risks to the public via exposure to these potentially dangerous creatures as well as creating pressure on wild snake populations [11]. The clinical effects of envenomation from these exotic species have been described more generally using broad descriptive terms [9]. Most often, the American Association of Poison Control Center designations for clinical outcomes such as mild, moderate or severe are utilized to describe data collected from these victims.

In an effort to provide a comprehensive picture of exotic snakebites reported to Pennsylvania’s two poison control centers, we critically analyzed exotic SBE cases from 2004 to 2018. The Pennsylvania poison control centers are based in Philadelphia and Pittsburgh and serve a state population of 12.7 million people. The Philadelphia Poison Control Center also services the State of Delaware with a population of approximately 1 million. However, no exotic SBE was reported from this state. Here, we present the demographics and clinical effects of exotic SBE cases as well as treatments used for the management of these victims.

## 2. Results

### 2.1. Original Data Extraction

A total of 1452 human exposure calls coded as ‘snakebite’ for the study period (2004–2018) were reported to the Pennsylvania Poison Control Centers, and 847 of these cases were subsequently identified as venomous snakebites. Out of these, 18 cases were determined to be non-native snakebites, and these were included in the analysis for this study (Table 1).

### 2.2. Patient Demographics

Of these 18 cases, a majority of the cases (15 of 18, 83%), were males. The mean age of the patients was 35 years (range from 16 to 63). Of note, three of the patients were also suspected of alcohol intoxication, although blood ethanol levels were not reported. One victim was reported to have been envenomed at a place of business, while the remaining were believed to have occurred within a private residence where the victim was either the owner of the exotic snake or an acquaintance of the owner. Of the 18 cases reported, one was suspected a dry bite as no clinical effects were observed, and one case was significant for an ocular exposure from an Equatorial spitting cobra (*Naja sumatrana*). In the case of a suspected dry bite from a specimen of *Naja kaouthia*, the patient was evaluated in the emergency department and left prior to the recommended observation period without returning. It is important to note that the absence of any symptoms at arrival to a health care facility does not necessarily mean that the patient has experienced a dry bite as symptoms may be delayed [12]. The fingers and hands were the most common sites of envenomation. However, one patient was bitten on the face and another on the buttocks.

### 2.3. Clinical Envenomation Effects

#### 2.3.1. Local Effects

**Figure 1:**
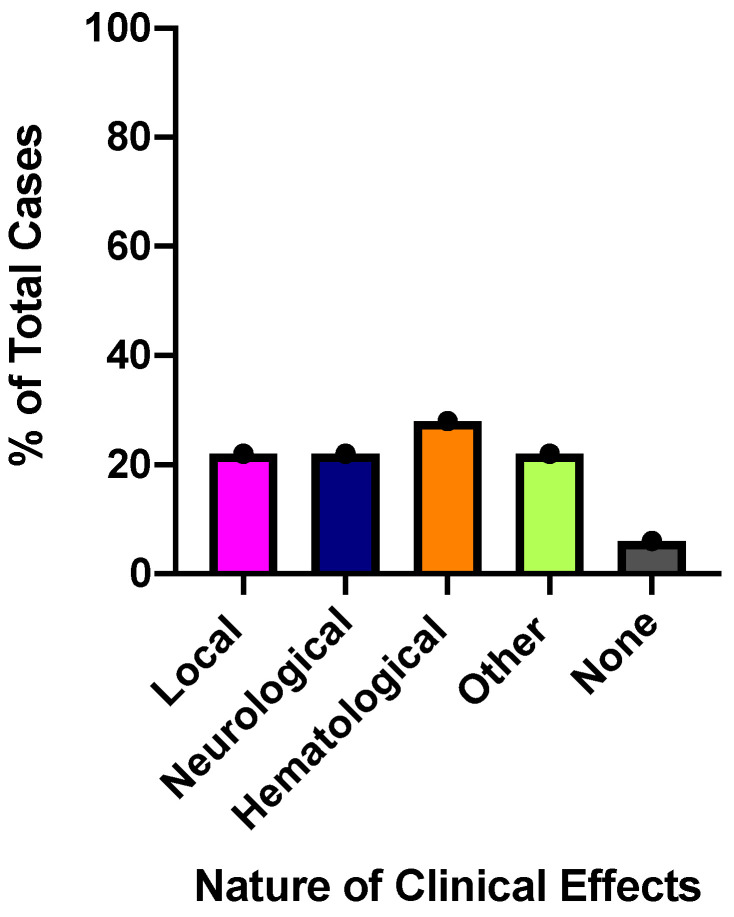
Different types of clinical effects observed in 18 cases of exotic snakebite envenomation (SBE) analyzed in this study. Some patients displayed multiple clinical effects.

From the exotic SBE cases reported, four described local tissue effects that ranged from mild redness at the bite site to diffuse edema and ecchymosis (Figure 1). The most significant local effects occurred as a result of a Puff adder (*Bitis arietans*) envenomation which occurred in the submandibular area. Diffuse ecchymosis and edema were reported extending from the bite site down to the neck and chest in this victim. The neck circumference measurement increased from 45 cm to 54 cm. This patient was endotracheally intubated due to the profound upper airway edema for over three weeks due to a suspected ventilator-associated pneumonia, which complicated their recovery. Blisters were also observed in two cases of *Trimeresurus* spp. envenomations and a bulla was seen in single patient with an *Atheris chlorechis* bite. The bulla was drained and one of the blisters underwent minor debridement, but no additional treatments were considered necessary for these local effects.

#### 2.3.2. Neurological Effects

Neurological effects were observed in four of 18 patients (22%) and included mild paresthesias as well as life-threatening bulbar and respiratory paralysis (Figure 1). Elapid envenomation resulted in the most significant neurological sequelae. An adult was bitten by both a juvenile and an adult Cape Coral cobra (*Aspidelaps lubricus*) while placing his hand inside the cage. This patient developed ptosis, dysphagia and upper extremity, facial, and respiratory paralysis, which resulted in endotracheal intubation and medical helicopter transport to the hospital. The Monocled cobra (*Naja kaouthia)* was responsible for four SBE in our study (as stated above one dry bite was also reported for this snake). Two of these patients underwent intubation due to bulbar involvement and respiratory paralysis. One patient, bitten on the buttocks remained asymptomatic for eight h then developed diplopia, dysphagia, and respiratory failure, and was therefore treated with mechanical ventilation. A single case of a viperid snake causing neurotoxic effects was also reported; the South American rattlesnake, *Crotalus durissus*, was involved in one bite to the finger of a 36-year-old patient, resulting in perioral paresthesia and diplopia.

#### 2.3.3. Hematological Effects

Changes in hematologic laboratory parameters were noted in five of 18 cases (28%) (Figure 1), however clinically significant bleeding was not reported in any of them. Thrombocytopenia was observed in two cases including one viperid, a Puff adder, *Bitis arietans* (platelets 84 × 10^9^/L; normal range (150–400) × 10^9^/L) and one elapid, a red spitting cobra, *Naja pallida* (platelets 102 × 10^9^/L). The *Naja pallida* envenomation also resulted in an elevated d-dimer of 3.63 µg/mL (normal <0.4 µg/mL) and a prolonged prothrombin time (PT) of 18.3 s (normal range 11–12.5 s). The single Puff adder envenomation resulted in an initial fibrinogen value of 249 mg/dL (normal range 200–400 mg/dL) that declined to a nadir of 104 mg/dL. One white-lipped pit viper bite, *Trimeresurus albolabris,* resulted in a modest fibrinogen decline from 224 to 185 mg/dL. Accurate timing of laboratory values was not readily available from the case information. Antivenom was administered in all cases reporting changes in hematologic laboratory values. Of the species causing hematological effects, all with the exception of *Naja pallida* are recognized to have the potential for clinically significant bleeding.

#### 2.3.4. Other Reported Clinical Effects

As mentioned above, one patient had an ocular exposure to a red spitting cobra and developed irritation and conjunctivitis that resolved with saline irrigation in the health care facility (Figure 1). No additional sequalae were observed in this patient. Pain at the site of envenomation was the most common complaint reported in all cases. An elevated creatine kinase (CK) level was noted in three of the cases. An envenomation from a Thai spitting cobra (*Naja siamensis*) caused a CK elevation of over 16,000 U/L (normal range 39–308 U/L), however, subsequent rhabdomyolysis was not observed. More modest CK elevations were noted in cases from *Crotalus durissus* (695 U/L) and *Naja pallida* (497 U/L).

### 2.4. Treatments Provided for Exotic SBE

**Figure 2:**
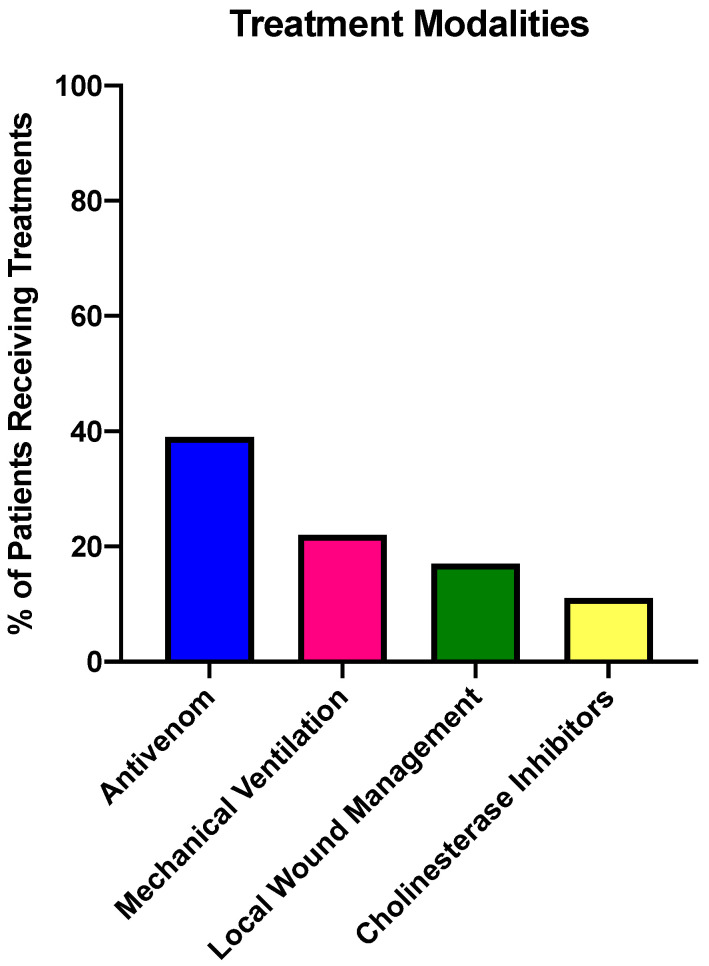
Nature of treatment provided for the exotic SBE cases reported in this study. Some patients received multiple interventions.

Antivenom was administered to seven of 18 patients (39%) within an average of four h from the time of envenomation (range three to nine h) (Figure 2). Time to antivenom administration data was available for five of the seven cases. The antivenom from South African Institute of Medical Research (SAIMR) was utilized in three cases, one viperid, *Bitis arietans*, and two elapids, *Naja siamensis* and *Naja kaouthia*. The polyvalent immune fab (CroFab^®^-BTG International) was administered in the single case of *Crotalus durissus* envenomation. The type of antivenom was not documented in three of the reported cases. The single patient treated with CroFab^®^(BTG) did not report any adverse effects, however two of the cases requiring SAIMR antivenom reported some minor shaking during administration. No serious adverse effects were noted. Early administration of antivenom is important and may result in faster limb recovery [13].

The cholinesterase inhibitors, pyridostigmine or neostigmine were given prior to antivenom in two of the reported *Naja kaouthia* envenomations (Figure 2), but the effectiveness was not noted. Mechanical ventilation was provided in four patients, three elapid bites, *Naja* spp *pallida*, *siamensis*, and *kaouthia* due to neurological effects and a viperid bite by *Crotalus durissus* due to edema as described above. Wound care was provided to patients as necessary, including incision and drainage of a bulla and debridement of several wounds. Pre-hospital measures were self-performed in three patients. Of these, one patient reportedly incised his site of envenomation and two patients used tourniquets applied to the affected areas. Incisions and tourniquets are not recommended and can actually worsen outcomes by causing serious damage including cellulitis or limb gangrene [14].

### 2.5. Time Spent in Health Care Facility

Following envenomation, patients arrived in hospitals either by private transport or via emergency medical services. Specific transport information was not available from the data reviewed. Two cases were admitted to a general medical-surgical floor for an average of 22 h. A total of seven patients were treated in the Intensive Care Unit (ICU). Time spent in the ICU averaged 124 h and ranged from 16 to 576 h. Of these patients, six received ICU care for less than five days, while a single patient with complications from a Puff adder bite remained for over three weeks. The remaining nine patients spent less than 24 h in the Emergency Department (ED).

## 3. Discussion

Exotic SBE has been reported in several regions of western countries. Envenomation may occur during handling or maintaining pet enclosures or other equipment. Due to reports of these bites in the USA, this study has identified and described detailed demographics, clinical effects and treatments as a result of exotic (non-native) snakebites reported to Pennsylvania Poison Control Centers for the 15-year period of 2004–2018. Of 1452 reported snakebite cases, a total of 18 (1.24%) were caused by snakes that are not native to North America. Clinical effects ranged from an asymptomatic patient from a presumed ‘dry bite’ to patients that received mechanical ventilation, one for several weeks due to complications.

Our work reported similar findings to a 2014 study that examined 258 exotic snakebites within the USA from 2005 to 2011 [9]. The mean age of patients in the present study was 35 years, including 83% males compared with a mean age of 33 years and 79% males in the national study. Antivenom was administered similarly with 39% receiving doses in this regional study versus 35% nationally. Adverse effects to antivenom were reported in 11% of our cases and these were generally mild in nature. This was higher than the reported 2.3% for the national study but far below the 20–80% that has been reported in some Asian studies [15]. Interestingly, *Naja kaouthia* accounted for the greatest number of SBE in both studies, perhaps reflecting greater availability, affordability, or reptile owner interest. This species was also noted to have been involved in a large number of SBE in a 1996 study that reported on non-native snakebites from 1977 to 1995 [16]. Bites from *Trimeresurus* spp, *Atheris* spp, and additional *Naja* species were the next most commonly reported exposures. 

The USA is not alone with regard to exotic SBE. A study that analyzed case inquiries to the United Kingdom National Poisons Information Service from 2004 to 2010 reported that 3% of all venomous snakebites in the UK were attributed to exotic species [17]. Similar to our data, the UK study reported that 69% of the cases were males with a mean age of 32 years of age. A poison center report from Hong Kong also describes several exotic snakebites [18]. A 34-year-old snake breeder in Switzerland was unknowingly envenomed by a black mamba (*Dendroaspis polyepis*) while feeding a specimen and a medical helicopter was used to transport antivenom to the treating hospital [19].

Elapid envenomation characteristically results in neurologic effects, whereas viper bites often present with hematological complications and local tissue injury. However, several of our cases demonstrate that clinical effects may overlap between these two families due to species-specific toxins [20]. For example, our case of the elapid, *Naja pallida* envenomation did report elevated prothrombin time and d-dimer level as well as thrombocytopenia. These findings reveal an anticoagulant effect that was recently demonstrated in a study of various cobra venoms [21]. Crotoxin is a component of the venom of the viperid South American rattlesnake, *Crotalus durissus* and is a potent phospholipase (PLA_2_) neurotoxin. The predominant effects of this toxin appear to be inhibition of neurotransmission at the pre-synaptic level. However, some post-synaptic effects are also possible [22,23]. Consistent with this toxin, our single case of *Crotalus durissus* envenomation reported both diplopia and paresthesias. While the majority of our cases suffered bites to the arms and hands, one patient was envenomed on the buttocks. This patient presented with delayed neurological effects eight h after the bite. The delay in onset of symptoms raises the question of possible slowed venom absorption due to site of envenomation, although we cannot rule out the possibilities of other factors including a minimal/diluted venom being injected.

Exotic snakebite is a rare occurrence in the USA and affords unique challenges to healthcare providers, many of whom have never cared for a snakebite victim previously. Interestingly, in areas where “exotic” species are endemic, such as Africa and parts of Asia, healthcare providers face obstacles in treatment and diagnosis as well, occasionally for different reasons. Frequently in the USA, exotic SBE occurs as a result of a reptile owner or acquaintance who has been bitten while handling the snake. In these cases, the victims typically know the species of snake since it has been purchased and cared for by the owner. In our case of a *Naja melanoleuca* bite, the owner had purchased antivenom prior to the envenomation and was able to bring the product with them to the hospital for treatment. Providers in many locations frequently have to deal with wild bites in which the offending snake is unable to be identified, thus complicating treatment decisions. These bites primarily occur in poor rural communities affecting agricultural workers, herders, fishermen, hunters, and inhabitants of improper housing. Antivenom procurement is a difficulty, which may be experienced by physicians and other providers both in the USA and abroad. In the USA, antivenom for exotic snakes is typically not stocked within hospitals but most often located at zoos, which are generous enough to make the product available for emergencies. An index of available antivenoms is available through the Association of Zoos and Aquariums and the School of Pharmacy at the University of Arizona. As these products are utilized very infrequently, many vials in the supply are outdated. Nevertheless, outdated products are used in situations where no alternatives are available. The antivenom may be located many miles from the treating facility, necessitating transport via helicopter as was necessary in two of our cases. Overseas providers face logistical challenges with antivenom as well. However, in many of these cases, health care facilities simply lack supply of the antivenom due to shortages or discontinued products [24]. The lack of appropriate antivenom for these exotic species is one of the major challenges in managing these bites.

Although antivenom is currently the only specific treatment utilized for SBE, there remain many difficulties surrounding its use. Procurement of antivenom can be challenging not only in rural areas of impoverished countries but also in cities of industrialized nations including the USA as well. Fortunately, newer non-antivenom therapeutics are being investigated, which may help to delay, prevent, or ameliorate the effects of envenomation, thus providing additional time for antivenom to be provided. Varespladib is a sPLA2 inhibitor that is being investigated for potential use as first-line treatment for SBE [21]. PLA_2_s are components of many venoms and produce significant pathological effects. Similarly, batimastat and marimastat are broad-spectrum matrix metalloprotease inhibitors which have been demonstrated to reduce the destructive effects of snake venom metalloproteases (SVMPs) [25,26]. A preliminary report of a combination of varespladib and marimastat, showed promise in an in vivo mouse model of SBE. This same study also reported the inhibition of a snake venom serine protease (SVSPs) by an inhibitor compound, nafamostat [27]. Additionally, other studies have investigated hyaluronidase inhibitors as potential agents for treating SBE [28,29,30].

The collection of reptiles and their maintenance is a popular hobby and many amateur collectors may easily obtain venomous species through the mail or by visiting reptile trade shows. Federal and state regulations can be ambiguous and the responsibility often falls to local government agencies to enact measures to control these potentially dangerous animals [31]. Even responsible, professional collectors are occasionally bitten, necessitating life-saving intervention. SBE is not only causing deaths, but also resulting in extensive skeletal muscle damage which frequently leads to permanent disabilities [32,33]. Moreover, SBE (specifically from vipers) can alter cardiovascular system, such as the function and number of platelets (as demonstrated in this study), which subsequently leads to thromboinflammatory complications [34,35,36]. Hence, it is critical to prevent SBE specifically from non-native species for which antivenom may not be readily available.

Our study is limited by the data within the National Poison Data System (NPDS) and the self-reporting by hospitals to poison control centers. As information is voluntarily reported, some clinical details may be incomplete or specific data may be subjectively reported by individuals caring for the patients. We were also limited by the lack of long-term follow-up which might enable us to track any prolonged injuries, side effects or permanent disabilities. In some cases, the details may be underreported as owners are fearful of legal repercussions and fail to seek medical assistance. Additional areas of future research might include a detailed cost analysis of treatments and long-term follow-up of patients to determine the extent of recovery. Despite these limitations, this study has highlighted the nature of exotic SBE among the developed communities, and the challenges they pose to clinicians during the clinical management of these cases. It is important that both the snake owners and relevant health authorities take into consideration potential SBE and the challenges in treating them while planning/permitting the purchase of such species.

## 4. Materials and Methods

For the initial data set, the NPDS database of the Philadelphia and Pittsburgh Pennsylvania Poison Control Centers, was searched from 2004 to 2018 using generic codes 0137000, and 0137102-013710. These internal codes are used to designate specific products, natural substances, animals, or other features relating to poisoning or envenomation. As described above, Delaware is included in the Philadelphia Poison Control Center service area. However, no exotic snakebites were reported from within this state. All 18 SBE described in this study were reported to have occurred within the State of Pennsylvania in the USA. This study was exempted from the Institutional Research Board for ethical considerations and only anonymized data were used. The de-identified records were abstracted. The demographic and clinical data were extracted from individual patient charts utilizing pre-defined criteria and placed into a spreadsheet. All the anonymized data were thoroughly checked for accuracy prior to analysis in this study.

## Figures and Tables

**Table 1:** Exotic snakebites by species, demographics, location, severity, and antivenom use.

Snake Species	Age (Years)	Gender	Location of Bite	Clinical Effects	Antivenom (Yes/No)
*Aspidelaps lubricus*	44	M	Hand	Severe	No
*Atheris chlorechis*	17	M	Finger	Unknown	No
*Atheris squamigera*	30	M	Finger	Moderate	No
*Bitis arietans*	50	F	Face	Severe	Yes *
*Bothrops schlegelii*	42	M	Finger	Minor	Yes
*Crotalus durissus terrificus*	36	M	Finger	Moderate	Yes
*Naja kaouthia*	23	F	Buttocks	Severe	Yes
*Naja kaouthia*	24	M	Forearm	Unknown	No
*Naja kaouthia*	31	M	Finger	None	No
*Naja kaouthia*	33	M	Unknown	Severe	Yes *
Naja kaouthia **	Unknown	M	Unknown	Unknown	No
*Naja melanoleuca*	63	F	Unknown	Minor	No
*Naja pallida*	16	M	Hand	Moderate	Yes *
*Naja siamensis*	38	M	Finger	Moderate	Yes *
*Naja sumatrana*	55	M	Ocular	Minor	No
*Trimeresurus albolabris*	24	M	Finger	Minor	No
*Trimeresurus albolabris*	28	M	Hand	Moderate	No
*Trimeresurus purpureomaculatus*	23	M	Finger	Moderate	No

** Suspected dry bite, * Antivenoms procured from Philadelphia zoo in these cases.
